# Supplementary material for: The gut microbiome but not the resistome is associated with urogenital schistosomiasis in preschool-aged children
Source: Commun Biol. 2020 Apr 2;3:155. doi: 10.1038/s42003-020-0859-7 (PMC7118151; doi:10.1038/s42003-020-0859-7)
Supplement: Supplementary file 1 — Supplementary Information [file 42003_2020_859_MOESM1_ESM.pdf]

Supplementary Figures

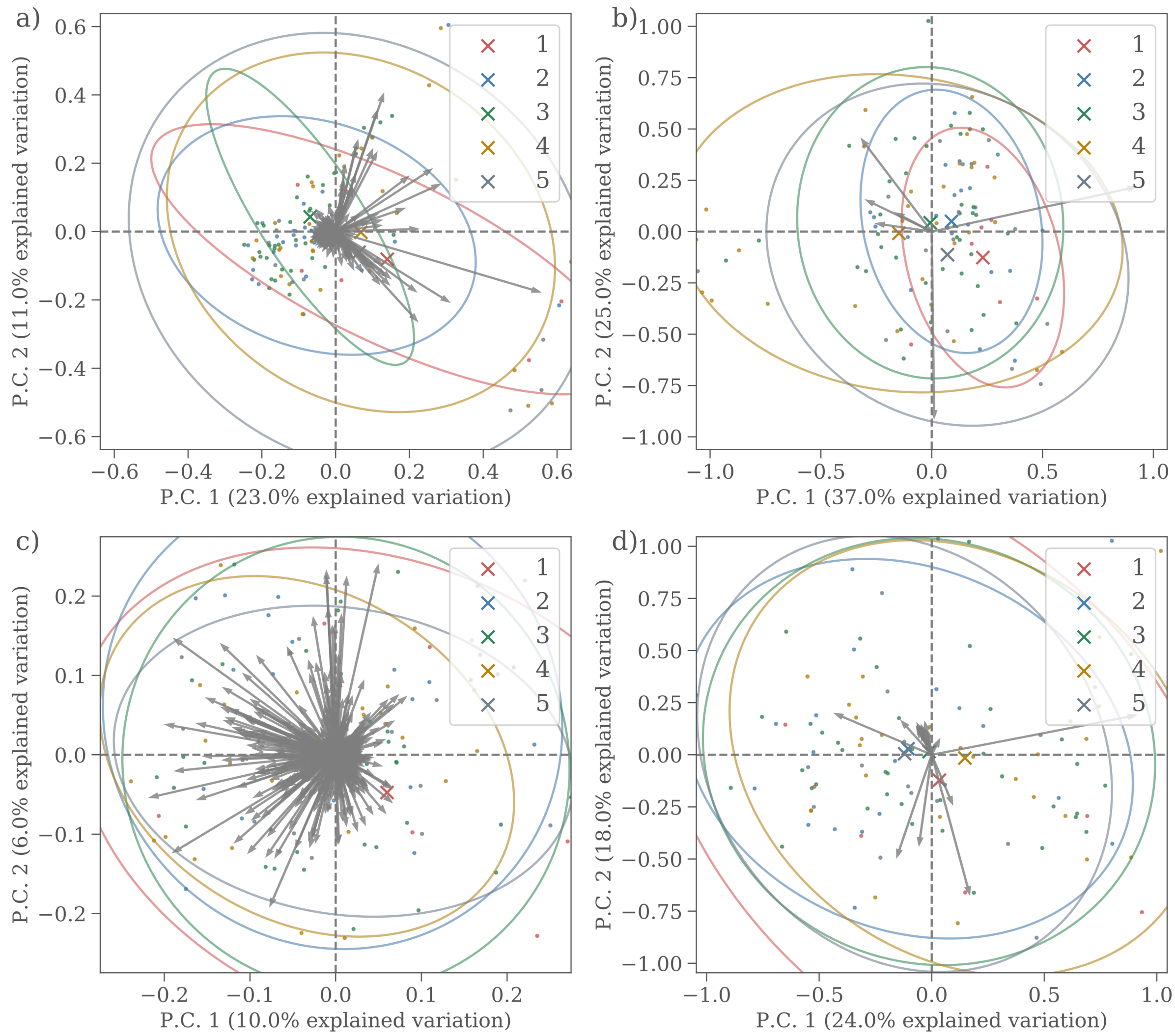

Supplementary Figure 1. Principal component analysis (PCA) plots for microbiota across samples, annotated by age. Figures a) and b) show PCA plots for fungi classified according to genus and phylum respectively. Figures c) and d) show PCA plots for bacteria classified according to genus and phylum respectively. Raw counts were *clr*-transformed and the resulting matrices were ordinated using PCA. The projection of features are plotted as arrows and the projection of samples are plotted as points. The eigenvalues associated with the eigenvectors are used to describe the amount of explained variation per axis.

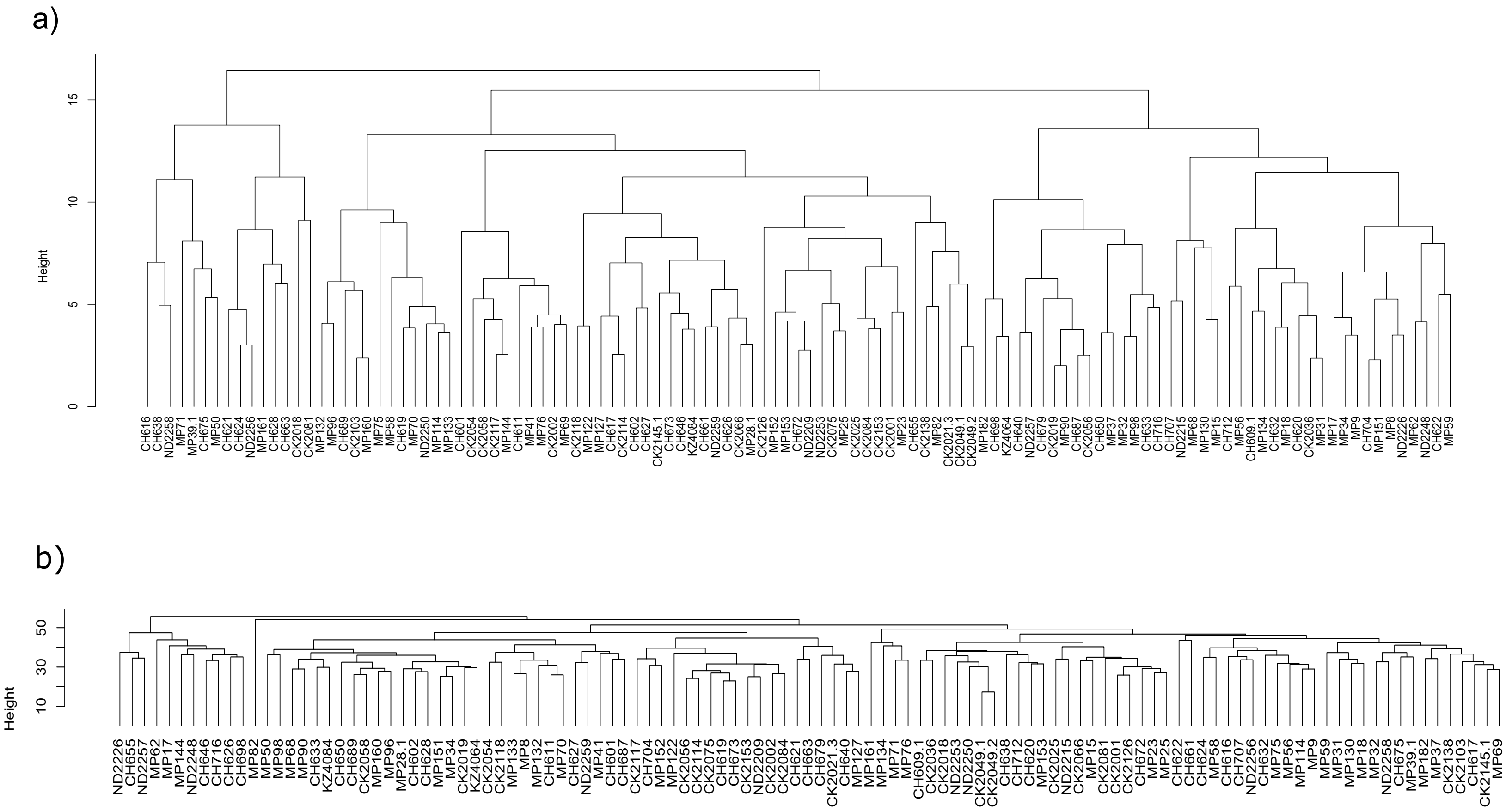

Supplementary Figure 2. Bacteria abundance and composition dendrograms. From read mapping to the genomic database, abundance was calculated for each microbial taxa across all samples. Clustered dendrograms show bacterial a) phyla and b) genera per sample. Raw counts were *clr*-transformed, the Aitchison distance (Euclidean distance) was calculated, and samples clustered based on distances (Complete-linkage-clustering).

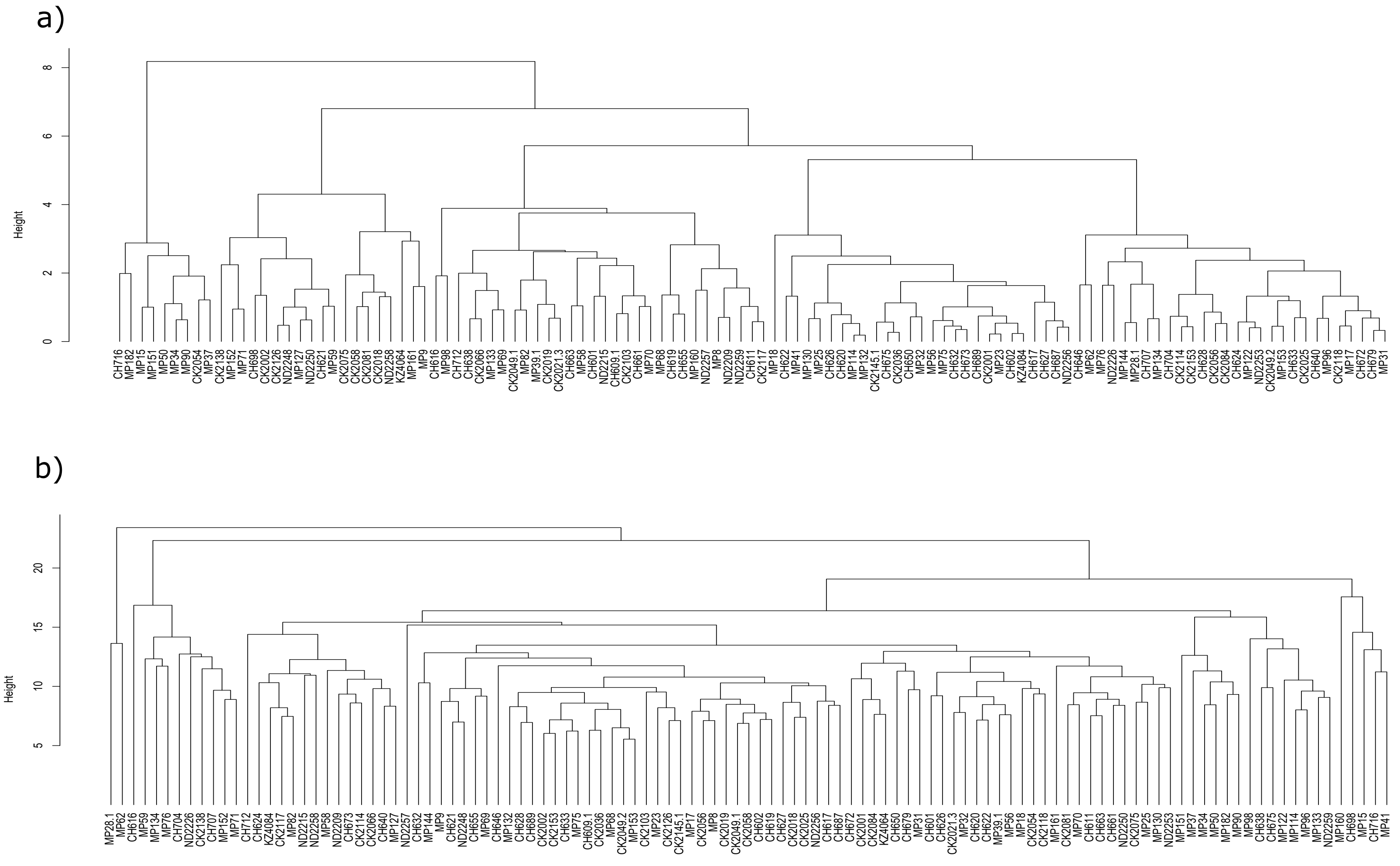

Supplementary Figure 3. Fungi abundance and composition dendrograms. From read mapping to the genomic database, abundance was calculated for each microbial taxa across all samples. Clustered dendrograms show fungal a) phyla and b) genera per sample. Raw counts were *clr*-transformed, the Aitchison distance (Euclidean distance) was calculated, and samples clustered based on distances (Complete-linkage-clustering).

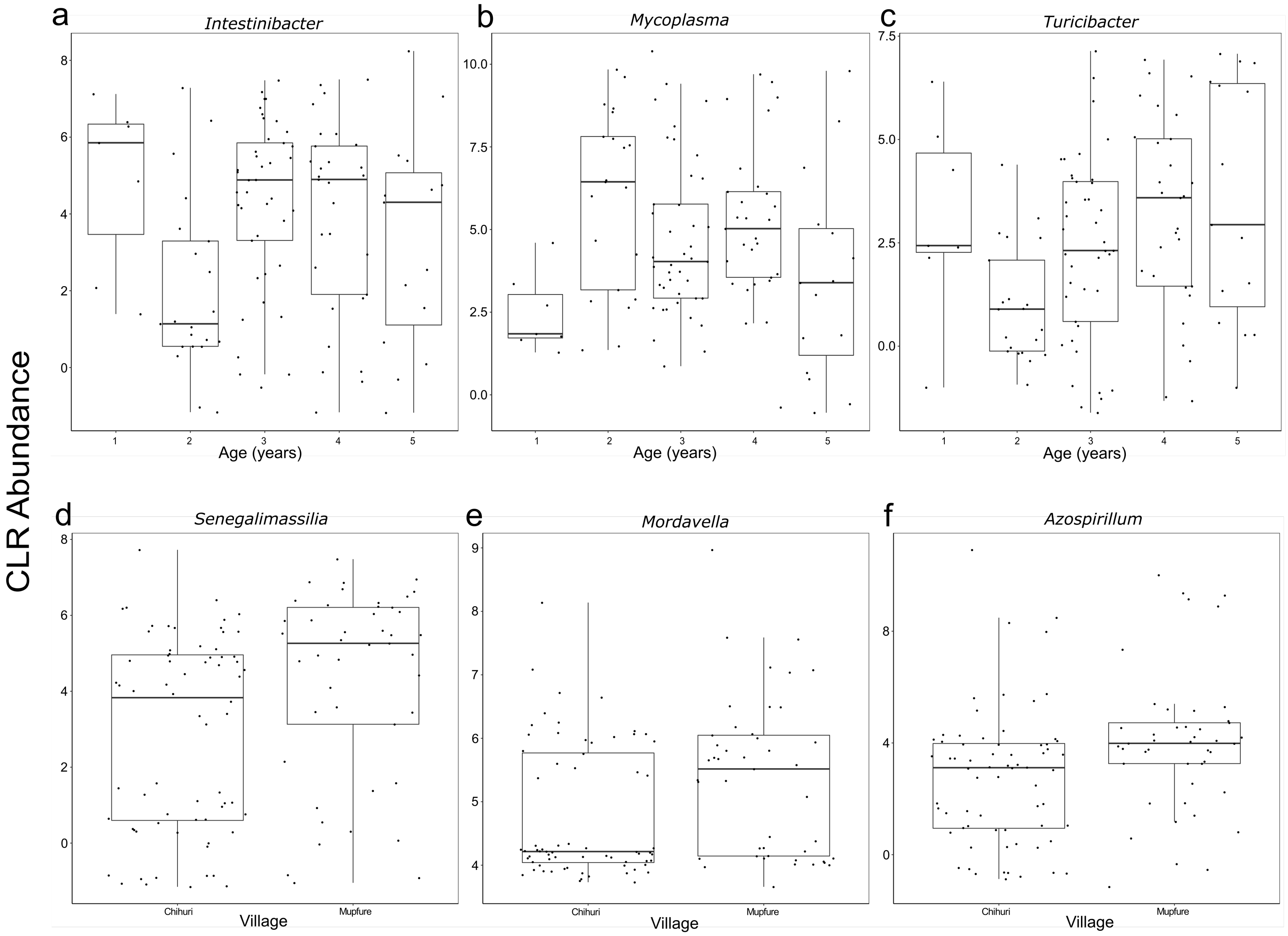

Supplementary Figure 4. Bacteria genera whose abundance vary significantly across host age and village. Figure a–c) Box plots showing the abundance of specific bacteria genera, grouped by age category in years. Figures d–f) Box plots showing the mean abundance of specific bacteria genera, grouped by village. Specific groups are represented on the x-axis and the abundances (*clr*-transformed) shown on the y-axis. The horizontal box lines represent the first quartile, the median and the third quartile. Whiskers denote the range of points within the first quartile − 1.5× the interquartile range and the third quartile + 1.5× the interquartile range. Specific bacteria genera were identified using analysis of composition of microbiomes (ANCOM).

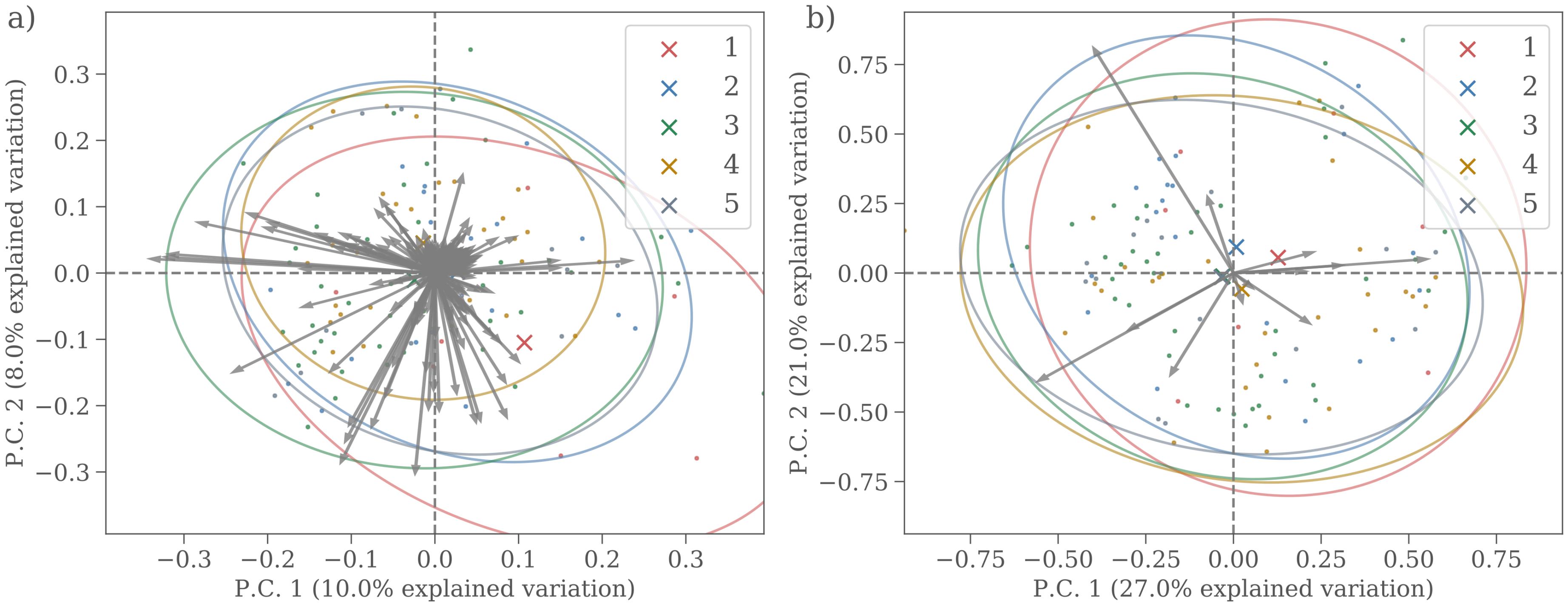

Supplementary Figure 5. Principal component analysis (PCA) plots for AMR genes and drug classes across samples, annotated by age. Figures a) and b) show PCA plots for AMR genes and AMR gene function classes respectively. Raw counts were *clr*-transformed and the resulting matrices were ordinated using PCA. The projection of features are plotted as arrows and the projection of samples are plotted as points. The eigenvalues associated with the eigenvectors are used to describe the amount of explained variation per axis.

a)

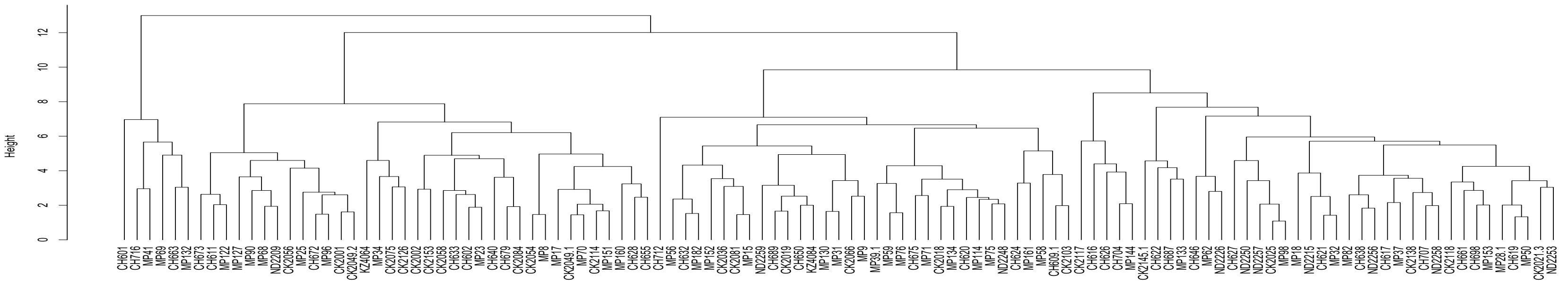

b)

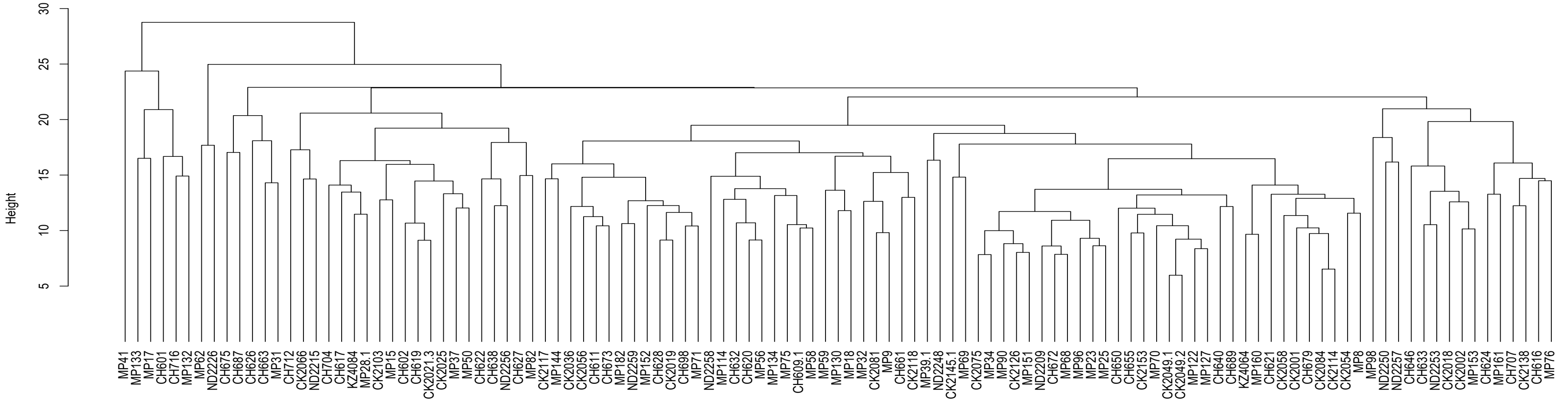

Supplementary Figure 6. AMR genes and drug class dendrograms. From read mapping to the ResFinder database, AMR abundance was calculated for each reference gene across all samples. Clustered dendrograms show AMR a) drug class and b) genes per sample. Raw counts were *alr*-transformed, the Aitchison distance (Euclidean distance) was calculated, and samples clustered based on distance (Complete-linkage-clustering).
