# Supplementary material for: The gut microbiome but not the resistome is associated with urogenital schistosomiasis in preschool-aged children
Source: Commun Biol. 2020 Apr 2;3:155. doi: 10.1038/s42003-020-0859-7 (PMC7118151; doi:10.1038/s42003-020-0859-7)
Supplement: Supplementary file 2 — Description of Additional Supplementary Files [file 42003_2020_859_MOESM2_ESM.pdf]

Description of Additional Supplementary Files

File Name: Supplementary Data 1

Description: All data and summary statistics generated and analysed from bioinformatics processing and mapping of sequence reads. This file also includes sample metadata. Each tab of the spreadsheet is labelled as appropriate to correspond with the content of the sheet.

File Name: Supplementary Data 2

Description: All source data generated from data analyses. This file includes raw sequence count data, abundance data and all *clr*-and *alr*- transformed data from which all statistical analyses and figures were produced. Each tab of the spreadsheet is labelled as appropriate to correspond with the content of the sheet

.
