# Supplementary material for: The gut microbiome but not the resistome is associated with urogenital schistosomiasis in preschool-aged children
Source: Commun Biol. 2020 Apr 2;3:155. doi: 10.1038/s42003-020-0859-7 (PMC7118151; doi:10.1038/s42003-020-0859-7)
Supplement: Supplementary file 5 — Reporting Summary [file 42003_2020_859_MOESM5_ESM.pdf]

nature research

Corresponding author(s): Derick Nii Mensah Osakunor; COMMSBIO-19-1562B

Last updated by author(s): Feb 10, 2020

Reporting Summary

Nature Research wishes to improve the reproducibility of the work that we publish. This form provides structure for consistency and transparency in reporting. For further information on Nature Research policies, see Authors & Referees and the Editorial Policy Checklist.

Statistics

For all statistical analyses, confirm that the following items are present in the figure legend, table legend, main text, or Methods section.

n/a	Confirmed	
☐	☒	The exact sample size (*n*) for each experimental group/condition, given as a discrete number and unit of measurement
☐	☒	A statement on whether measurements were taken from distinct samples or whether the same sample was measured repeatedly
☐	☒	The statistical test(s) used AND whether they are one- or two-sided<br>*Only common tests should be described solely by name; describe more complex techniques in the Methods section.*
☐	☒	A description of all covariates tested
☐	☒	A description of any assumptions or corrections, such as tests of normality and adjustment for multiple comparisons
☐	☒	A full description of the statistical parameters including central tendency (e.g. means) or other basic estimates (e.g. regression coefficient) AND variation (e.g. standard deviation) or associated estimates of uncertainty (e.g. confidence intervals)
☐	☒	For null hypothesis testing, the test statistic (e.g. *F*, *t*, *r*) with confidence intervals, effect sizes, degrees of freedom and *P* value noted<br>*Give P values as exact values whenever suitable.*
☒	☐	For Bayesian analysis, information on the choice of priors and Markov chain Monte Carlo settings
☒	☐	For hierarchical and complex designs, identification of the appropriate level for tests and full reporting of outcomes
☐	☒	Estimates of effect sizes (e.g. Cohen's *d*, Pearson's *r*), indicating how they were calculated

Our web collection on statistics for biologists contains articles on many of the points above.

Software and code

Policy information about availability of computer code

Data collection	No Software was used
Data analysis	Microsoft R Open 3.3.2 (R distribution with library version control and multi-threading support)<br>RStudio 1.0.136 (GUI for R)<br>vegan 2.4.1 (Community Ecology R Package)<br>KMA (k-mer alignment): https://cge.cbs.dtu.dk/services/KMA/ or https://bitbucket.org/genomicepidemiology/kma<br>ANCOM (Analysis of Composition of Microbiomes: a methodology to detect differentially abundant taxa in microbial surveys): https://sites.google.com/site/siddharthamandal1985/research OR https://github.com/zellerlab/crc_meta/blob/master/src/ANCOM_updated.R

For manuscripts utilizing custom algorithms or software that are central to the research but not yet described in published literature, software must be made available to editors/reviewers. We strongly encourage code deposition in a community repository (e.g. GitHub). See the Nature Research guidelines for submitting code & software for further information.

Data

Policy information about availability of data

All manuscripts must include a data availability statement. This statement should provide the following information, where applicable:

- Accession codes, unique identifiers, or web links for publicly available datasets
- A list of figures that have associated raw data
- A description of any restrictions on data availability

Raw sequence data files from all 116 samples and associated metadata used in the current study are deposited in the Sequence Read Archive (SRA) of the National Centre for Biotechnology Information (NCBI) database under the BioProject accession number PRJNA521455. All other data are available on request to the corresponding author.

Field-specific reporting

Please select the one below that is the best fit for your research. If you are not sure, read the appropriate sections before making your selection.

☒ Life sciences ☐ Behavioural & social sciences ☐ Ecological, evolutionary & environmental sciences

For a reference copy of the document with all sections, see nature.com/documents/nr-reporting-summary-flat.pdf

Life sciences study design

All studies must disclose on these points even when the disclosure is negative.

Sample size	The samples used in the current study are from the baseline survey of a larger epidemiological study comparing re-infection rates across two different treatment regimens. As this is a relatively new field in human helminthology, there are limited published studies, with none focusing on the age group in the current study, i.e. 1-5 year olds. Thus there were no published baseline data to inform sample size calculations when we conducted our study. The sample size for the current study was informed by our previous study (Kay, Millard et al. 2015) and those of others (Schneeberger, Coulibaly et al. 2018, Ajibola, Rowan et al. 2019) in older children with sample sizes ranging from 34–139, from which significant differences were detected in the microbiome of schistosome infected versus uninfected children.
Data exclusions	Three (3) metagenomic datasets were excluded for the overall analysis using a predefined exclusion criteria (final n=113). To appropriately explain variations in the data, samples with non-missing data from at least one variable metadata from growth and nutrition, schistosome infection status, previous schistosome treatment and antibiotic use data, were used for all downstream analysis.
Replication	No replication was performed in the classical sense but duplicate samples collected from two (2) participants were used as biological and technical replicates for shotgun metagenomic sequencing. Analysis, confirmed clustering and similarity in their sequence components.
Randomization	This was a cross-sectional study using baseline samples from the larger study and samples were selected by convenient random sampling from participants who provided consent for the present study, based on predefined criteria.
Blinding	This was a cross-sectional study using baseline samples from the larger study and thus blinding was not applicable to this study. Library preparation and shotgun sequencing was done by BGI in China, who were blind to all metadata associated with samples. The field team were also blinded from any metadata associated with samples selected. No attempts were made at blinding during DNA extraction and data analysis.

Reporting for specific materials, systems and methods

We require information from authors about some types of materials, experimental systems and methods used in many studies. Here, indicate whether each material, system or method listed is relevant to your study. If you are not sure if a list item applies to your research, read the appropriate section before selecting a response.

Materials & experimental systems

n/a	Involved in the study	
☒	☐	Antibodies
☒	☐	Eukaryotic cell lines
☒	☐	Palaeontology
☒	☐	Animals and other organisms
☐	☒	Human research participants
☒	☐	Clinical data

Methods

n/a	Involved in the study	
☒	☐	ChIP-seq
☒	☐	Flow cytometry
☒	☐	MRI-based neuroimaging

Human research participants

Policy information about studies involving human research participants

Population characteristics	113 participants included in final analysis (mean age was 3.7±1.1 years, of which 56 were female (49.6%), 68 (60.2%) were from Chihuri and 45 (39.8%) from Mupfure villages. S. haematobium infection prevalence was 15.9% (18/113), with mean infection intensity of 1.79 eggs/10 ml urine (SEM= 0.76; range= 0–74).
Recruitment	As part of the larger study, participants who gave consent for their samples to be used as part of this study were recruited based on a specified criteria; a) consent for microbiome analysis, b) availability of socio-demographic data, c) availability of parasitology samples (urine and stool samples), d) availability of test results and clinical history, and e) no current episode of diarrhoea (assessed by questionnaire and visual stool examination).
Ethics oversight	Ethics: Medical Research Council of Zimbabwe (MRCZ/A/1964)<br>Institutional approval : University of Edinburgh (fmutapi-0002)

Note that full information on the approval of the study protocol must also be provided in the manuscript.
